# Occupational exposure to pesticides deregulates systemic cortisol levels in women with breast cancer and correlates with poor prognosis features

**DOI:** 10.1590/1414-431X2023e13060

**Published:** 2024-01-22

**Authors:** J.J. Jumes, H.S. Jaques, M.F. Dalla Vecchia, M.O. Ferreira, J.F.G. Orrutéa, M.G. Machado, M.F. Mezoni, R.G.S. da Silva, R.F. Almeida, D. Rech, A.C.B. Kawassaki, C. Panis

**Affiliations:** 1 Laboratório de Biologia Tumoral, Universidade Estadual do Oeste do Paraná, Francisco Beltrão, PR, Brasil; 2 Programa de Ciências da Saúde Aplicadas, Universidade Estadual do Oeste do Paraná, Francisco Beltrão, PR, Brasil; 3 Hospital de Câncer de Francisco Beltrão, CEONC, Francisco Beltrão, PR, Brasil

**Keywords:** Breast cancer, Cortisol, Pesticides, Endocrine disruption

## Abstract

Pesticides have been pointed out as hormone disruptors and may significantly affect the prognosis of hormone-dependent diseases such as breast cancer (BC). Here, we investigated the impact of occupational pesticide exposure on systemic cortisol levels in female rural workers diagnosed with BC. Occupational exposure was assessed by interviews with a standardized questionnaire. Plasma samples (112 from pesticide-exposed women and 77 from unexposed women) were collected in the afternoon, outside the physiological cortisol peak, and analyzed by a chemiluminescent paramagnetic immunoassay for the quantitative determination of cortisol levels in serum and plasma. The results from both groups were categorized according to patients' clinicopathological and exposure data. BC pesticide-exposed women presented higher levels of cortisol than the unexposed. Higher cortisol levels were also detected in the exposed group with more aggressive disease (triple-negative BC), with tumors over 2 cm, with lymph node metastases, and with high risk of disease recurrence and death. These findings demonstrated that there is an association between pesticide exposure and BC that affected cortisol levels and correlated to poor disease prognosis.

## Introduction

Breast cancer (BC) is the most common malignant neoplasia in women worldwide. In 2020, 2.3 million women were diagnosed with BC, and a total of 685,000 died from the disease (1). Although the main risk factors related to BC are age, family history, and reproductive and hormonal factors, environmental exposures play a significant role in BC (2,3). The negative impact of pesticide exposure has been extensively associated with BC risk (4-6) and is linked to significant immune deregulation (7,8) and DNA repair impairment (9).

Exposure to pesticides has systemic impacts. Widely used pesticides, such as glyphosate and atrazine, are known endocrine disruptors (10,11) and may significantly affect the course of hormone-dependent diseases such as BC. They can act as hormone deregulators by affecting hormone synthesis, secretion, transport, binding, action, or elimination (12). A variety of pesticides can mimic estrogen, and because of this, they can act as xenoestrogens. Further, pesticides have been detected in adipose tissue samples from BC women (13), suggesting accumulation in the mammary gland. Consequently, exposure to such endocrine disruptors can affect breast cell division and differentiation (14,15), pivotal events linked to BC initiation and progression.

One of the less explored hormones in BC biology is cortisol, a reliable biomarker of endocrine deregulation of the hypothalamic-pituitary-adrenal (HPA) axis (16,17). Studies point out that pesticides can significantly alter the cortisol axis in BC. For example, patients with abnormal cortisol secretion present enhanced tumor progression and immune system malfunctioning (18,19).

Pesticides also affect the production of inflammatory mediators such as Th1 cytokines (15). This pro-inflammatory environment leads to genomic instability, resulting in DNA damage and increased risk of BC development (20). Chronic systemic inflammation also favors the deregulation of the cortisol axis by making it hyperactivated (21). Cortisol has a physiological anti-inflammatory action but can cause tumor growth and progression under dysregulated conditions (22).

Patients with metastatic BC and continuously increased systemic cortisol levels tend to present early mortality (23), suggesting that the combination of deregulated cortisol and BC may result in a poor prognosis. Here, we hypothesized that women exposed to pesticides have cortisol imbalance, and that this is associated with worse disease compared to unexposed women. Considering this, we investigated the impact of occupational pesticide exposure on the systemic cortisol profile of BC patients. We measured cortisol levels in blood samples from pesticide-exposed and unexposed BC patients outside the physiological cortisol peak, and analyzed the results according to the main clinicopathological features determinant of BC prognosis.

## Material and Methods

The present study screened 422 women who were under investigation for BC at Francisco Beltrão Cancer Hospital (CEONC), a public hospital in the southwest of Paraná state (Brazil) that assists 27 municipalities. A total of 182 women with confirmed BC from May 2015 to August 2022 and clinicopathological data available were included in the study. This was a mixed, observational, analytical, cross-sectional cohort study approved by the Institutional Ethics and Human Research Committee (Opinion CAAE number 72169517.1.0000.0109). All participants signed a free and informed consent form.

To understand the impact of pesticide exposure on cortisol levels, we collected heparinized peripheral blood samples (10 mL) in the afternoon (2-5 pm), outside the morning cortisol peak, to understand whether cortisol levels are deregulated during the day. Samples were centrifuged at 5600 *g* for 5 min at 4°C, and plasma was frozen at -20°C for later analysis. Cortisol levels were measured by a chemiluminescent cortisol immunoassay using the UNICEL DXI 800 equipment (Beckman Coulter, USA).

**Figure 1:**
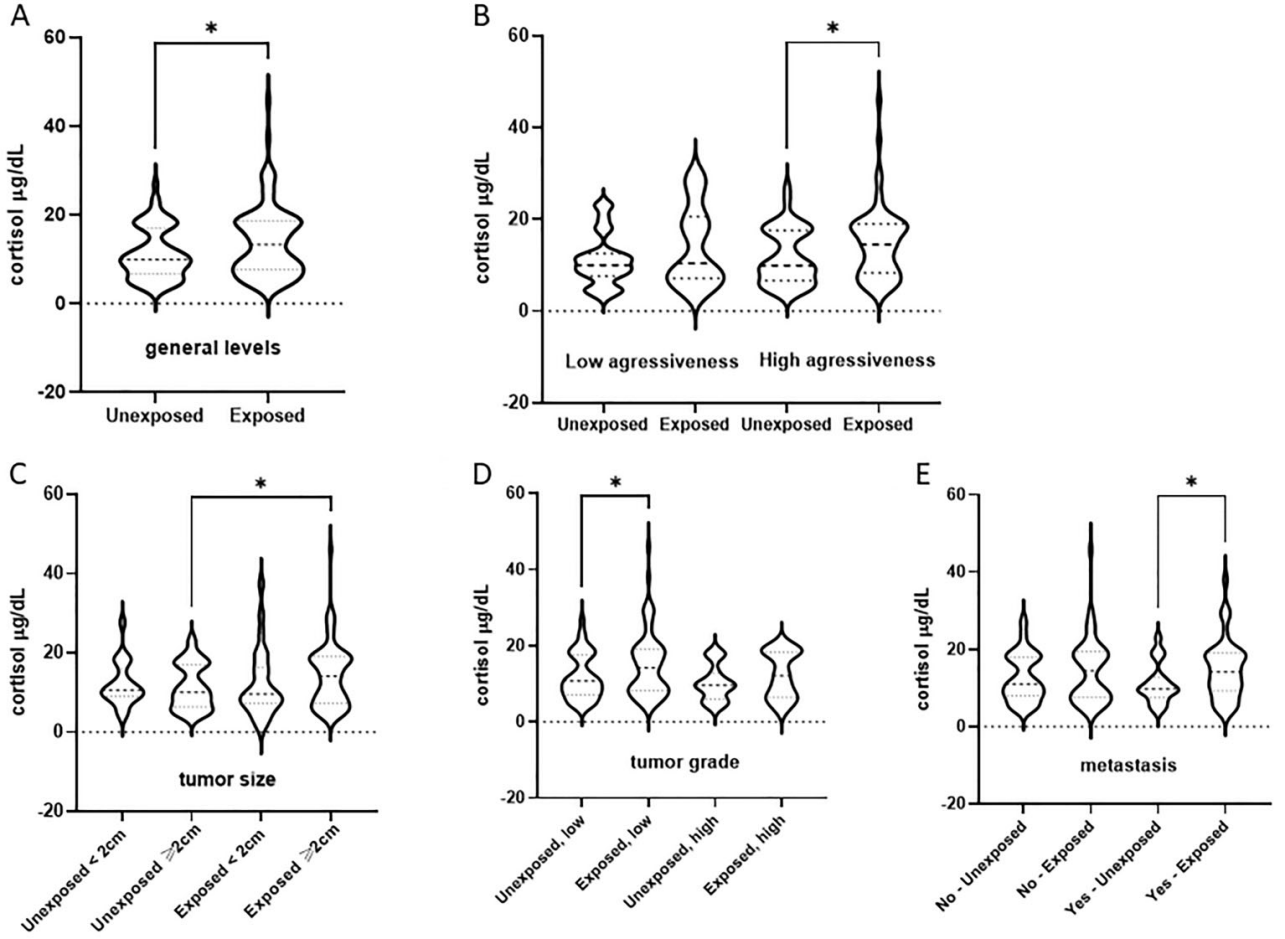
Cortisol levels in plasma samples from breast cancer patients exposed or not to pesticides categorized according to their clinicopathological features. **A**, overall circulating levels; **B**, cortisol levels according to disease aggressiveness (low=luminal tumors, high=triple-negative breast cancer); **C**, cortisol levels according to tumor size (2 cm cut-off); **D**, cortisol levels according to tumor grade (low=1 and 2, high=3); and **E**, cortisol levels according to presence or absence of metastasis. Data are reported as violin plots. *P<0.05, Mann-Whitney's test. The dotted line represents the start of the axis (level zero).

Clinicopathological information from BC patients was collected from medical records, which included age at diagnosis, histological grade, tumor size, lymph node metastasis, menopausal status at diagnosis, body mass index (BMI), and molecular subtype. For clinicopathological characterization (24), samples were grouped according to disease aggressiveness into low (Luminal A) *vs* high (HER2-amplified, Triple-negative +, or Luminal B), tumor size (< or ≥2 cm), tumor grade (low-grade=1 and 2, high-grade=3), and disease metastasis (yes or no) (Figure 1).

Patients were also interviewed to assess their occupational profile concerning pesticide exposure. To reach this goal, we used a validated instrument (25) containing questions about their past and present occupational activities concerning pesticide handling. To identify present and past occupational exposure to pesticides, the team used 60 questions focusing mainly on the pesticide type and duration of exposure, how women were contaminated (handling, spraying, washing clothes), and putative intoxications. The exposure criteria were based on continuous, unprotected, and direct handling of pesticides. Women working in rural areas with direct contact with pesticides (pesticide preparation and dilution, spraying, or washing/decontaminating clothes and protection equipment), and who reported being in contact with pesticides at least 50% of their lives at least twice a week during all weeks of the year were considered exposed. The unexposed group consisted of women who reported never being occupationally exposed to pesticides and urban workers with no previous or current history of occupational exposure to pesticides.

Central tendency and dispersion measures were used for descriptive analyses. Data distribution was tested using the Shapiro-Wilk test, and variables with normal distribution were analyzed using parametric tests. When the assumption of normality was not met, non-parametric tests were used. The Student's *t*-test was used to compare parametric data, and the Mann-Whitney test was used to compare non-parametric data. Data were analyzed using the GraphPad Prism 9.0 software (USA), and a P-value <0.05 was considered significant.

## Results

Clinicopathological data from BC patients are shown in Table 1. The explored variables were molecular subtype, tumor size, histological grade, lymph node metastasis, age at diagnosis, menopausal status at diagnosis, and BMI. BC patients exposed to pesticides had significantly more metastases than unexposed ones (P<0.05).

**Table 1:** Clinicopathological data from breast cancer patients included in the study categorized according to pesticide exposure.

Variable / Group	Category	Percent	P value
Estrogen receptor			
Exposed	Negative	35.71	0.079
	Positive	64.29	
Unexposed	Negative	18.18	
	Positive	81.82	
Progesterone receptor			
Exposed	Negative	48.08	0.755
	Positive	51.92	
Unexposed	Negative	51.61	
	Positive	48.39	
Ki67 (%)			
Exposed	<14	96.23	0.305
	>14	3.77	
Unexposed	<14	90.91	
	>14	9.09	
Molecular subtype			
Exposed	Luminal A	34.69	0.780
	Luminal B	54.08	
	HER2	1.02	
	Triple negative	10.21	
Unexposed	Luminal A	40.32	
	Luminal B	50.00	
	HER2	0.00	
	Triple negative	9.68	
Tumor size			
Exposed	<2	25.89	0.586
	>2	74.11	
Unexposed	<2	29.58	
	>2	70.42	
Histological grade			
Exposed	Grade 1	32.71	0.369
	Grade 2	46.73	
	Grade 3	20.56	
Unexposed	Level 1	30.99	
	Level 2	39.44	
	Level 3	29.58	
Metastasis lymph node			
Exposed	No	51.85	0.003*
	Yes	48.15	
Unexposed	No	73.08	
	Yes	26.92	
Age at diagnosis			
Exposed	≤ 50	45.70	0.650
	> 50	54.30	
Unexposed	≤50	48.57	
	> 50	51.42	
Menopausal status			
Exposed	Yes	51.16	0.568
	No	48.84	
Unexposed	Yes	46.15	
	No	53.85	
Body mass index			
Exposed	Obese	39.08	0.746
	Eutrophic	60.92	
Unexposed	Obese	41.82	
	Eutrophic	58.18	

*P<0.05, Fisher's exact test.

Figure 1 shows cortisol levels in plasma samples from exposed and unexposed BC patients according to the different clinicopathological conditions that determine breast cancer prognosis and behavior. Plasma cortisol levels were significantly higher in the exposed group (45.7±0.8 μg/dL) compared to the unexposed group (Figure 1A, 27.6±0.9 μg/dL, P=0.0085). Cortisol levels were also higher in exposed BC patients with highly aggressive tumors compared to the unexposed group (Figure 1B, 45.7±0.8 μg/dL for BC exposed + high aggressiveness and 19.6±5.21 μg/dL for BC exposed + low aggressiveness, P=0.0093).

BC patients exposed to pesticides and with tumors larger than 2 cm had higher cortisol levels than the unexposed group (Figure 1C, 26.9±0.9 μg/dL and 5.7±0.8 μg/dL, respectively, P=0.0087). Regarding tumor grade (Figure 1D), higher levels of cortisol were detected in exposed patients with high-grade tumors in relation to those with low-grade BC (45.7±0.85 μ/dL and 29.5±0.8 μ/dL, respectively, P=0.0407). In addition, plasma cortisol levels were higher in the exposed BC patients with metastasis (Figure 1E, 37.3±0.8 μg/dL) than in unexposed metastatic BC patients (23.1±0.9 μg/dL).

Table 2 shows the correlation between cortisol levels according to the risk stratification of death and recurrence in BC patients occupationally exposed or not to pesticides. As demonstrated, cortisol levels were significantly higher in BC women exposed to pesticides with a high risk of death and recurrence than in unexposed patients. In Table 3, cortisol levels are shown according to age at diagnosis and BMI in the two groups. No significant differences were found among groups.

**Table 2:** Cortisol levels according to risk stratification of death and recurrence in patients with breast cancer patients exposed or not to pesticides.

Risk stratification	Unexposed	Exposed	P-value
Low risk	12.01±0.95	12.57±1.53	0.4505
Intermediate risk	12.67±1.05	13.17±1.17	0.7594
High risk	10.72±1.03	15.55±1.25	0.0092*

*P<0.05, Student's *t*-test.

**Table 3:** Cortisol levels according to age at diagnosis and body mass index (BMI) in breast cancer patients exposed or not to pesticides.

Parameter	Unexposed	Exposed	P-value
Age <50 years	12.51±0.83	13.46±1.21	0.8442
Age ≥50 years	11.79±0.82	13.89±0.96	0.2994
Eutrophic BMI	12.49±0.87	13.32±1.21	0.8887
Obese BMI	11.89±0.80	14.01±0.99	0.3833

BMI: body mass index. Student's *t*-test.

## Discussion

The present study found that chronic and continued pesticide exposure deregulated systemic cortisol levels in BC women, and was associated with poor prognosis. Although the literature concerning cortisol levels in BC patients is scarce and there are no data on pesticide exposure, changes in cortisol levels correlate with early mortality and poor disease prognosis in cancer patients (26). As far as we know, this is the first study addressing the relationship between pesticide exposure, cortisol deregulation, and disease prognosis in BC patients.

The comparative analysis showed that BC women exposed to pesticides had higher circulating cortisol levels than unexposed BC patients, indicating the deregulation of the cortisol axis, since physiological levels are low at the end of the day. Increased levels of pesticides are reported in the plasma of BC patients (27), suggesting that chronic exposure can lead to its constant accumulation in the body. Considering that altered cortisol is a marker of poor prognosis in BC (28) and pesticides are known deregulators of this axis (29), we further investigated cortisol levels according to disease features that are determinants of disease prognosis.

BC prognosis mainly depends on the molecular subtype (30). We demonstrated that BC patients with aggressive tumors occupationally exposed to pesticides had higher cortisol levels. The mechanisms by which pesticides are involved in cancer pathophysiology encompass modifications of gene expression related to proliferation (31). We found significant cortisol changes with tumor size and grade. These changes also occur according to BC subtypes, and the most clinically aggressive tumors, such as triple-negative BC, are usually high-grade and can be very proliferative (32).

Moreover, the more aggressive the tumor, the higher the chance to develop metastasis. Our findings suggested a relationship between BC, pesticide exposure, and cortisol. We found a significantly higher frequency of metastasis in the BC-exposed group and higher levels of cortisol in the pesticide-exposed patients with metastasis. It is suggested that such immunological suppression, among other factors, may be influenced by increased systemic cortisol levels in patients exposed to pesticides, which seems to contribute to metastatic BC and worse clinical outcomes (33). These data are very worrisome because the leading cause of death in cancer patients is not the primary tumor, but the occurrence of metastasis (34).

Despite no evidence about the relationship between cortisol, pesticides, and BC metastasis, the known mechanisms link these events. Inflammation is a leading event in cancer progression and co-exists with cortisol changes in cancer patients (19), leading to metastasis in BC (35). We have previously demonstrated that occupational exposure of BC women to pesticides leads to deregulated inflammation (8). Therefore, the hypothesis that deregulation of both cortisol and the immune response to pesticides affects metastasis is plausible and may have implications for the spreading of BC, as demonstrated here.

This fact has a direct implication on the clinical fate of BC patients. Because of this, we analyzed cortisol levels according to the risk of death and disease recurrence in such patients and found higher cortisol in BC-exposed women categorized as high risk. Occupational pesticide exposure can be a modifier of BC behavior, and several previous studies support this. The authors of one study compared occupationally exposed and unexposed BC patients with intermediate-risk diseases and found many immune-related impairments that occurred only in the exposed group (7). The risk of death is linked to the presence of lymph node metastases and other BC risk factors (36-39). These findings suggest that intermediate-risk BC patients with pesticide exposure could be evaluated as having high-risk disease behavior.

This study had limitations, including modest sample size, lack of multiple cortisol analyses in a time-dependent manner, and follow-up about disease recurrence and survival. Despite this, the study clearly demonstrated that there was a relationship between pesticide exposure and BC that affected cortisol levels and correlated to poor disease prognosis.

## References

[1] IARC (International Agency For Research On Cancer) Cancer today. Lyon: WHO, 2020. https://gco.iarc.fr/today/home.

[2] Sun YS, Zhao Z, Yang ZN, Xu F, Lu HJ, Zhu ZY (2017). Risk factors and preventions of breast cancer. Int J Biol Sci.

[3] Feng Y, Spezia M, Huang S, Yuan C, Zeng Z, Zhang L (2018). Breast cancer development and progression: Risk factors, cancer stem cells, signaling pathways, genomics, and molecular pathogenesis. Genes Dis.

[4] Duell EJ, Millikan RC, Savitz DA, Newman B, Smith JC, Schell MJ (2000). A population-based case-control study of farming and breast cancer in North Carolina. Epidemiology.

[B05] Brophy JT, Keith MM, Gorey KM, Laukkanen E, Hellyer D, Watterson A (2002). Occupational histories of cancer patients in a Canadian cancer treatment center and the generated hypothesis regarding breast cancer and farming. Int J Occup Environ Health.

[6] Louis LM, Lerro CC, Friesen MC, Andreotti G, Koutros S, Sandler DP (2017). A prospective study of cancer risk among Agricultural Health Study farm spouses associated with personal use of organochlorine insecticides. Environ Health.

[7] da Silva JC, Scandolara TB, Kern R, Jaques HDS, Malanowski J, Alves FM (2022). Occupational exposure to pesticides affects pivotal immunologic anti-tumor responses in breast cancer women from the intermediate risk of recurrence and death. Cancers (Basel).

[8] Pizzatti L, Kawassaki ACB, Fadel B, Nogueira FCS, Evaristo JAM, Woldmar N (2020). Toxicoproteomics Disclose pesticides as downregulators of TNF-α, IL-1β and estrogen receptor pathways in breast cancer women chronically exposed. Front Oncol.

[9] Scandolara TB, Valle SF, Teixeira CE, Scherer NM, de Armas EM, Furtado C (2022). Somatic DNA damage response and homologous repair gene alterations and its association with tumor variant burden in breast cancer patients with occupational exposure to pesticides. Front Oncol.

[10] Atoum MF, Alzoughool F, Al-Hourani H (2020). Linkage between obesity leptin and breast cancer. Breast Cancer (Auckl).

[11] Hokanson R, Fudge R, Chowdhary R, Busbee D (2007). Alteration of estrogen-regulated gene expression in human cells induced by the agricultural and horticultural herbicide glyphosate. Hum Exp Toxicol.

[12] Rocha MJ, Cruzeiro C, Rocha E (2013). Quantification of 17 endocrine disruptor compounds and their spatial and seasonal distribution in Iberian Ave river and its coastline. Toxicol Environ Chem.

[13] Rutkowska AZ, Szybiak A, Serkies K, Rachoń D (2016). Endocrine disrupting chemicals as potential risk factor for estrogen-dependent cancers. Pol Arch Med Wewn.

[14] Tapia-Orozco N, Santiago-Toledo G, Barrón V, Espinosa-García AM, García-García JA, García-Arrazola R (2017). Environmental epigenomics: current approaches to assess epigenetic effects of endocrine disrupting compounds (EDC's) on human health. Environ Toxicol Pharmacol.

[15] Filippi I, Carraro F, Naldini A (2015). Interleukin-1β affects MDAMB231 breast cancer cell migration under hypoxia: role of HIF-1α and NFκB transcription factors. Mediators Inflamm.

[16] Thompson PA, Khatami M, Baglole CJ, Sun J, Harris SA, Moon EY (2015). Environmental immune disruptors, inflammation and cancer risk. Carcinogenesis.

[17] Ockenfels MC, Porter L, Smyth J, Kirschbaum C, Hellhammer DH, Stone AA (1995). Effect of chronic stress associated with unemployment on salivary cortisol: overall cortisol levels, diurnal rhythm, and acute stress reactivity. Psychosom Med.

[18] Zhang L, Pan J, Chen W, Jiang J, Huang J (2020). Chronic stress-induced immune dysregulation in cancer: implications for initiation, progression, metastasis, and treatment. Am J Cancer Res.

[19] Schrepf A, Clevenger L, Christensen D, DeGeest K, Bender D, Ahmed A (2013). Cortisol and inflammatory processes in ovarian cancer patients following primary treatment: relationships with depression, fatigue, and disability. Brain Behav Immun.

[20] Kawanishi S, Ohnishi S, Ma N, Hiraku Y, Murata M (2017). Crosstalk between DNA Damage and Inflammation in the multiple steps of carcinogenesis. Int J Mol Sci.

[21] Giulivo M, de Alda ML, Capri E, Barceló D (2016). Human exposure to endocrine disrupting compounds: Their role in reproductive systems, metabolic syndrome and breast cancer. A review. Environ Res.

[22] Weiser MJ, Handa RJ (2009). Estrogen impairs glucocorticoid dependent negative feedback on the hypothalamic-pituitary-adrenal axis via estrogen receptor alpha within the hypothalamus. Neuroscience.

[23] Sephton SE, Sapolsky RM, Kraemer HC, Spiegel D (2000). Diurnal cortisol rhythm as a predictor of breast cancer survival. J Natl Cancer Inst.

[24] Buitrago F, Uemura G, Sena MCF (2011). Fatores prognósticos em câncer de mama. Com Ciên Saúde.

[25] Panis C, Gaboardi SC, Kawassaki ACB, Dias ECM, Teixeira GT, Silva DRP (2022). Characterization of occupational exposure to pesticides and its impact on the health of rural women. Rev Bras Cien Farm.

[26] Shin KJ, Lee YJ, Yang YR, Park S, Suh PG, Follo MY (2016). Molecular mechanisms underlying psychological stress and cancer. Curr Pharm Des.

[27] Mekonen S, Ibrahim M, Astatkie H, Abreha A (2021). Exposure to organochlorine pesticides as a predictor to breast cancer: a case-control study among Ethiopian women. PLoS One.

[28] Pan D, Kocherginsky M, Conzen SD (2011). Activation of the glucocorticoid receptor is associated with poor prognosis in estrogen receptor-negative breast cancer. Cancer Res.

[29] Zhang X, Zhong Y, Tian H, Wang W, Ru S (2015). Impairment of the cortisol stress response mediated by the hypothalamus-pituitary-interrenal (HPI) axis in zebrafish (*Danio rerio*) exposed to monocrotophos pesticide. Comp Biochem Physiol C Toxicol Pharmacol.

[30] Rakha EA, Green AR (2017). Molecular classification of breast cancer: what the pathologist needs to know. Pathology.

[31] Porreca I, D'Angelo F, De Franceschi L, Mattà A, Ceccarelli M, Iolascon A (2016). Pesticide toxicogenomics across scales: *in vitro* transcriptome predicts mechanisms and outcomes of exposure *in vivo*. Sci Rep.

[32] Cancer Genome Atlas Network (2012). Comprehensive molecular portraits of human breast tumours. Nature.

[33] Fernandes JV, Cobucci RN, Jatobá CA, Fernandes TA, de Azevedo JW, de Araújo JM (2015). The role of the mediators of inflammation in cancer development. Pathol Oncol Res.

[34] Dillekås H, Rogers MS, Straume O (2019). Are 90% of deaths from cancer caused by metastases?. Cancer Med.

[35] Herrera RA, Deshpande K, Martirosian V, Saatian B, Julian A, Eisenbarth R, Das D (2022). Cortisol promotes breast-to-brain metastasis through the blood-cerebrospinal fluid barrier. Cancer Rep (Hoboken).

[36] Zhang W, Xu J, Wang K, Tang XJ, Liang H, He JJ (2020). Independent risk factors for axillary lymph node metastasis in breast cancer patients with one or two positive sentinel lymph nodes. BMC Womens Health.

[B37] Tonellotto F, Bergmann A, Abrahão KS, de Aguiar SS, Bello MA, Thuler LCS (2019). Impact of number of positive lymph nodes and lymph node ratio on survival of women with node-positive breast cancer. Eur J Breast Health.

[B38] Riggio AI, Varley KE, Welm AL (2021). The lingering mysteries of metastatic recurrence in breast cancer. Br J Cancer.

[39] Koual M, Tomkiewicz C, Cano-Sancho G, Antignac JP, Bats AS, Coumoul X (2020). Environmental chemicals, breast cancer progression and drug resistance. Environ Health.

